# Effect of Probenecid on Endothelial Cell Growth Rate and Retinal Angiogenesis in an Oxygen-Induced Retinopathy Model

**DOI:** 10.3389/fphar.2021.717351

**Published:** 2021-10-06

**Authors:** Jingbo Jiang, Weiming Ou, Xianqiong Luo, Jianwen Xiang, Guosheng Liu, Shuiqing Huang, Hongping Li, Longkai He, Jiamin Gan, Shasha Han, Chuan Nie

**Affiliations:** ^1^ Neonatology Department, Shenzhen Children’s Hospital, Shenzhen, China; ^2^ Department of Pediatrics, The First Affiliated Hospital, Jinan University, Guangzhou, China; ^3^ Neonatology Department, Guangdong Women and Children Hospital, Guangzhou, China; ^4^ Department of Neonatology and Pediatrics, The First Affiliated Hospital, Jinan University, Guangzhou, China; ^5^ Neonatology Department, Guangdong Women and Children’s Hospital, Guangzhou Medical University, Guangzhou, China

**Keywords:** CMAP, retinopathy of prematurity (ROP), hepcidin (HAMP), retinal neovascularization (RNV), vascular endothelial cells (ECs), oxygen-induced retinopathy (OIR)

## Abstract

**Objectives:** Probenecid is an anion transport inhibitor, which, according to the connectivity map (CMap; a biological application database), interferes with hypoxia-induced gene expression changes in retinal vascular endothelial cells (ECs). Here, we investigated the influence of probenecid on retinal EC cytotoxicity and retinal neovascularization in a murine oxygen-induced retinopathy (OIR) model.

**Methods:** The retinal EC growth rate in the presence of hypoxia-mimicking concentrations of cobalt chloride (CoCl_2_) was determined using the thiazolyl blue tetrazolium bromide (MTT) assay and proliferating cell nuclear antigen (PCNA) expression. In OIR rats, probenecid was administered by intraperitoneal injection (i.p.) from postnatal day (P) 1 to P7. The concentrations of vitreous humor vascular endothelial growth factor (VEGF), hypoxia-inducible factor (HIF)-1α, and placental growth factor (PlGF) were determined by using the ELISA kit at P21. The amount of newly formed vascular lumen was evaluated by histopathological examination. Retinopathy and neovascularization were assessed by scoring isolectin B4 fluorescein–stained retinal flat mounts. Western blots for liver tissue HIF-1α and hepcidin (HAMP) were performed.

**Results:** *In vitro*, probenecid led to the recession of the hypoxia-induced EC growth rate. *In vivo*, compared to the OIR retina, the upregulation of VEGF, HIF-1α, and PlGF in phase II retinopathy of prematurity (ROP) was inhibited by probenecid administration. Moreover, probenecid ameliorated neovascularization and resulted in significantly reduced relative leakage fluorescence signal intensity in fluorescein-stained retinal flat mounts (*p* < 0.05). Probenecid alleviated the liver overactivation of HAMP and downregulation of HIF-1α in OIR rats.

**Conclusions:** This is the first demonstration that implies that probenecid might be a protective compound against retinal angiogenesis in OIR. These changes are accompanied with decreased hyperoxia-mediated hepcidin overproduction. Although the relevance of the results to ROP needs further research, these findings may help establish potential pharmacological targets based on the CMap database.

## Introduction

Despite improved oxygen monitoring and better neonatal practice, retinopathy of prematurity (ROP) remains a major cause of blindness and visual handicap in children globally, occurring almost exclusively among infants with a birth weight <1,250 g. Among all premature infants undergoing ROP screening examinations in a large representative cohort at multiple centers, 3,224 (43.1%) developed ROP and approximately 12.5% developed severe ROP (Quinn et al., 2018). Treated ROP is a strong independent predictor of later neurodevelopmental impairment in childhood and adolescence (Schmidt et al., 2015). The balance between optimal oxygen supplementation to survive versus minimal oxygen to avoid retinal vasoproliferation has not been classified for prematurity at different gestational and postmenstrual ages (Raghuveer and Zackula, 2020).

The treatment of ROP is evolving from mechanical (laser or cryotherapy) to biological agents. Successful treatment remains a key research area and depends on multiple factors and prompts diagnosis. Currently, laser photocoagulation or cryotherapy for the peripheral retina remains the standard care, but these interventions destroy the avascular retina and are often inefficient with zone I ROP. Tailored therapies to reduce aberrant vasoproliferation and facilitate physiological retinal vascular development and neurovascular interaction without harming the developing infant are needed. In recent years, the VEGF antibody (bevacizumab) has shown encouraging results in decreasing the need for additional laser ablation (Hartnett, 2020; Twitty et al., 2020; Wallace et al., 2020). Other biological agents that are currently being studied include IGF-1 with IGF-binding protein-3 (rhIGF-1 + rhIGFBP-3), omega-3 long-chain polyunsaturated fatty acids (LCPUFAs), D-penicillamine, propranolol, and antioxidants (Qureshi and Kumar, 2013; Hellstrom et al., 2017; Malamas et al., 2017; Ley et al., 2019).

The immature retina is metabolically active and susceptible to variations in oxygen tension. The oxygen supply–demand disequilibrium along with other stress responses, postnatal nutrition deficit, and lack of growth factors result in disordered intravitreal neovascularization. Many signal proteins and cytokines are implicated in the process of neovascularization, such as vascular endothelial growth factor (VEGF), non–oxygen-regulated growth factor insulin-like growth factor-1 (IGF-1), cyclooxygenase-2, growth hormone, PlGF, and hypoxia-inducible factor 1 (HIF-1) (Lazzara et al., 2020). Using microarray analysis, our previous studies have revealed that genes involved in the iron homeostasis pathway such as hepcidin antimicrobial peptide (HAMP) are highly enriched under hypoxic conditions (Luo et al., 2015). The liver secretes hepcidin to reduce blood iron levels by triggering the degradation of the only known cellular iron exporter ferroportin (Fpn). The systemic dysregulation of the HAMP/Fpn axis leads to retinal iron accumulation and degeneration (Theurl et al., 2016). It is well-established that iron accumulates in the retina and causes oxidative damage and significant morphologic and structural changes, compared to those found in age-related macular degeneration (AMD) and diabetic retinopathy (Baumann et al., 2019; Picard et al., 2020), indicating that these genes are implicated in the development of ROP as well. However, direct evidence to support the role of hepcidin in the pathogenesis of ROP is still lacking.

Based on the differentially expressed genes identified in hypoxic settings, we used the CMap database, a bioinformatics-validated strategy that identifies new applications for established drugs, to search and screen for molecule compounds that interfere with iron homeostasis. The results showed that probenecid is capable of reverting the gene expression profile of pathological phenotypes in retinal ECs suffering oxygen deprivation. We next sought to evaluate the effects of probenecid on retinal angiogenesis in a newborn OIR rat model. A model of fluctuations in oxygenation in newborn rats first recreates the peripheral avascular retina, followed by vasoproliferation at the junction of the vascularized and avascular retina, similar to human ROP. To test this hypothesis, we investigated whether probenecid influences retinal levels of proangiogenic factors, retinal vascularization, and central vaso-obliteration. It is well-established that there is a molecular link between hepatic hepcidin and intestinal HIF-α that controls physiological iron uptake and drives iron hyperabsorption during iron overload (Schwartz et al., 2019). A portion of prematurity received red blood transfusion; as a result, iron overload is common in this population. Therefore, hepcidin and HIF-1α expressions in the liver were evaluated to help understand whether changes in systemic hepcidin are related to retinal neovascularization disease.

## Materials and Methods

### Connectivity Map Query

Differentially expressed genes (DEGs) involved in hypoxia-raised fatal retinal ECs were assessed using an Agilent Sureprint G3 Human GE 8 × 60 K Microarray kit (Agilent Tech. Santa Clara, CA, United States), as previously reported (Luo et al., 2015). The one-class algorithm was applied to screen the DEGs with a threshold set at a fold change > 2.0 and a q-value < 0.05. A total of 326 gene symbols were separated into those that were upregulated (198 genes) or downregulated (128 genes) to generate a connectivity map (Luo et al., 2015). In the present study, 326 differentially expressed genes were transformed into query signature format files and put in the CMap website (http://www.broad.mit.edu/cmap/). After comparing with the built-in gene expression profiles, probenecid is identified according to the intensity of the connectivity score, indicating that it has an opposite relationship with ROP.

### Cell Culture and Treatments

Human retinal ECs were obtained from Dr. Lei (Sun Yat-sen University) and cultured, as described previously (Xiaozhuang et al., 2010). In brief, the cells were maintained on fibronectin-coated culture dishes in PTI-MEM I reduced serum medium (Gibco) with 10% fetal bovine serum (Hyclone Logan, UT, United States), 1% insulin–transferrin–selenium (ITS, Gibco), 25 ng/ml β-endothelial cell growth factor (R&D, Minn., United States), 100 μg/ml streptomycin, and 100 U/ml penicillin at 37°C in a humidified incubator containing 5% CO_2_, with the medium changed every alternate day. The cells were serum-starved for 4 h prior to probenecid (SYNCO Ltd, Hong Kong) treatment (150 μg/ml), and 150 μmol cobalt chloride (Sigma) was used to mimic the hypoxic condition for 2 h. The cell viability tests were conducted as previously described (Zhang et al., 2009).

### Animals

We acknowledge the ethical principles of Frontiers in Pharmacology and confirm that the protocols were performed in accordance with these principles and the Institutional Animal Care and Use Committee at Guangzhou Medical University (GWCH-XSE-12001). The *in vivo* experiments were performed on wild-type Sprague Dawley (S-D) albino rats of both sexes. The rats were reared in a pathogen-free facility with a 12-h light/dark cycle and *ad libitum* access to food and water. For probenecid treatment, half of the pups, depending on each experimental design, from each litter received probenecid [1 mg/kg body weight dissolved in distilled H_2_O (dH_2_O)] intraperitoneally once a day from P1 to P7. For the control group, sham injections were administered with an equivalent volume of dH_2_O. At P22, the rats were deeply anesthetized by intraperitoneal injection of 0.35 ml avertin (2.5% tribromoethanol, Sigma-Aldrich) for future experiments.

### Oxygen-Induced Retinopathy (OIR) Model

Individual litters were reared in either oxygen or room air. For the OIR model, litters of SD rat pups with their nursing mothers were exposed to 75% oxygen between postnatal day P1 and P14 to induce retinal vessel loss. After exposure, the rats were placed in room air for an additional 7 days. The oxygen concentration was checked twice daily using an oxygen analyzer.

### Enzyme-Linked Immunosorbent Assay (ELISA)

At P22, retinal tissue was harvested, resuspended in 1:20 w/v T-PER tissue protein extraction reagent (Thermo Scientific, 78,510), and disrupted in a chilled tissue homogenizer. The resulting lysates were centrifuged at 15,000 × *g* for 5 min. The supernatant was collected and transferred into tubes containing protease inhibitors. The protein concentration of each lysate was determined using the BCA protein assay (Pierce, 23,227). The release of VEGF-A, HIF-α, and PlGF was quantitatively examined from the respective medium samples using a commercial ELISA system (Invitrogen, MA). Samples and standards were prepared in duplicate, and each group contained nine independent samples. The plate was analyzed using a microplate spectrophotometer at a wavelength of 450 nm.

### Paraffin Sections and Hematoxylin and Eosin (H&E) Staining

Rat eyeballs were fixed in 4% paraformaldehyde overnight, dehydrated, embedded in paraffin wax, and serially sectioned parallel to the optic nerve at 5 µm. For histological examination, the sections were de-waxed in xylene, rehydrated, and stained with H&E. The sections were photographed using an upright microscope (Olympus BX53, Tokyo, Japan).

### Retinal Flat Mount Preparation and Isolectin Fluorescence Staining

After being anesthetized, the eyes were enucleated and fixed in 4% paraformaldehyde solution for 2 h. The retinas were dissected, isolated intact, and fixed in 4% PFA for another 10 min. Each retina was evenly cut into four pieces to form a “four-leaf clover” shape, with the optic papilla as the center. The tissue was soaked in 0.3% Triton X-100 for 20 min and blocked with 10% fetal calf serum. Next, isolectin GS-IB4 (Invitrogen, I21414) (2 μM) was added, and the retina was incubated overnight at 4°C. The retina was rinsed three times with phosphate-buffered saline (PBS) and coverslipped. The relative area of vessel obliteration and fluorescence leakage was quantified using a confocal laser fluorescence microscope (LSM 510 META, Carl Zeiss, Germany) that was linked to cellSens Standard 1.9 software.

### Quantitative Assessment of Retinal Neovascularization

For retinal sagittal sections stained with H&E, the vascular lumen extending into the vitreous humor was calculated. Ten non-continuous sections from each eye (each 20 µm apart) were examined, and the average number of pre-retinal vascular lumens in each group was compared. In the isolectin-stained retinal flat mount, the total area of the retina was traced according to the outermost vessel of the arcade as the border. The tuft area was first selected manually and then measured by adjusting the threshold in ImageJ software (National Institute of Health, MD) based on the high intensity of isolectin staining.

### Quantitative Assessment of Central Vaso-Obliteration

The area of central vascular obliteration was outlined and measured using ImageJ software (National Institute of Health, MD); this area and the total retinal area were computed, and the percentage of the central vascular obliteration area over the total retinal area was calculated.

### Western Blotting

Western blot analysis was performed on the proteins extracted from liver samples under each experimental condition. In brief, the liver tissues were physically homogenized and lysed in M-PER Mammalian Protein Extraction Reagent (Thermo Fisher, MA, 78,503) and centrifuged at 22,000 × *g* for 30 min at 4°C. The supernatant was used for the detection of HAMP or β-actin. The protein concentration was measured according to the Bradford method using bovine serum albumin (BSA) as a standard. The protein samples from all related experiments were processed in lithium dodecyl sulfate sample loading buffer (Bio-Rad, CA, 1,610,737), heated at 95°C for 5 min, loaded onto 10% Tris–glycine stain–free gel (Bio-Rad, 5,678,033), resolved by SDS-PAGE, and transferred to a polyvinylidene difluoride membrane using a Turbo Transfer System (Bio-Rad, 1,704,155).Specific antibodies directed to distinct HAMP (1:1,000; ThermoFisher, MA, PA5-90884) were used. The signals were detected by horseradish peroxidase (HRP)–based chemiluminescence (ThermoFisher, MA, 34,095), exposed to ECL Chemidoc (Bio-Rad, 1,708,280), and digitized using Image Lab software (Bio-Rad, 1,709,692).The methods have been described in detail in our earlier articles.

### Statistical Analyses

Statistical analysis was performed using GraphPad Prism 6 for Windows (GraphPad Software, San Diego, CA, United States).A two-tailed unpaired t-test was used for comparison between the two groups. Data expressed as mean ± SD were analyzed by Student’s t-test. For the analysis of groups more than two, one-way ANOVA or the Kruskal–Wallis test will be chosen depending on the homogeneity of variance. Statistical significance was set at a *p* value < 0.05.

## Results

### Probenecid Inhibits CoCl_2_-Mediated Retinal EC Cytotoxicity

**FIGURE 1:**
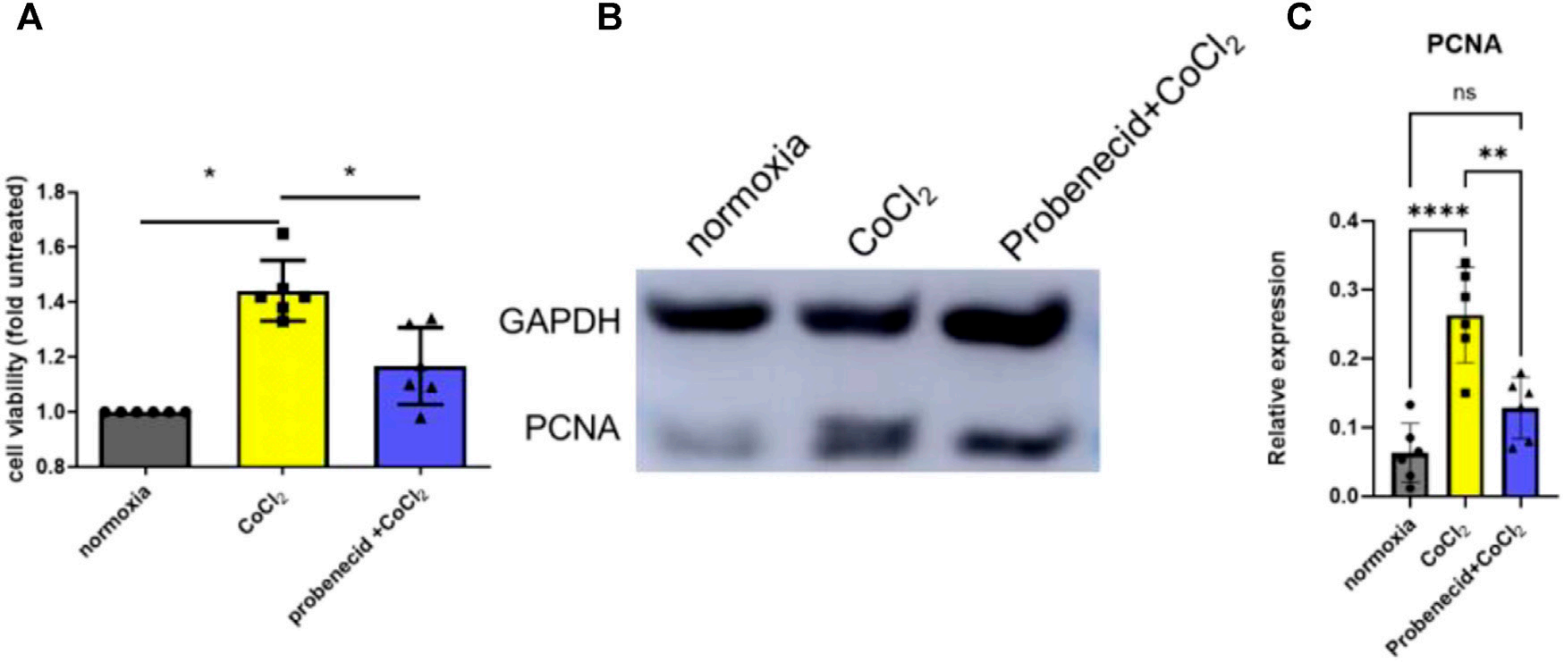
Probenecid inhibits CoCl_2_-mediated retinal EC proliferation. Cells were pretreated with and without probenecid (150 μg/ml) for 60 min prior to CoCl_2_ exposure. MTT assay was conducted in human retinal ECs to analyze cell viability. **(A)** Bar graphs represent fold untreated of cell viability compared with normoxic conditions. **(B)** Representative WB for PCNA expression. **(C)** Bar graphs represent the relative PCNA level in three conditions. Results are expressed as mean ± SD; n = 6 for each group, *p < 0.05, **p < 0.0015, and ****p < 0.0001. Significance was determined by one-way ANOVA with Tukey’s post hoc test.

Previous studies have shown that the retinal EC growth rate is increased when (150 μM) CoCl_2_, a hypoxic mimetic reagent, was introduced into the medium (Zhang et al., 2009). Here, we demonstrated that pretreatment with probenecid (150 μg/ml) before CoCl_2_ exposure significantly reduced the retinal EC viability in comparison to the hypoxia group (Figure 1).The elevated PCNA expression after CoCl_2_ exposure was attenuated by probenecid pretreatment.

### Overproduction of VEGF, HIF-1α, and PlGF in Phase II ROP Was Prevented by Probenecid Pretreatment

**FIGURE 2:**
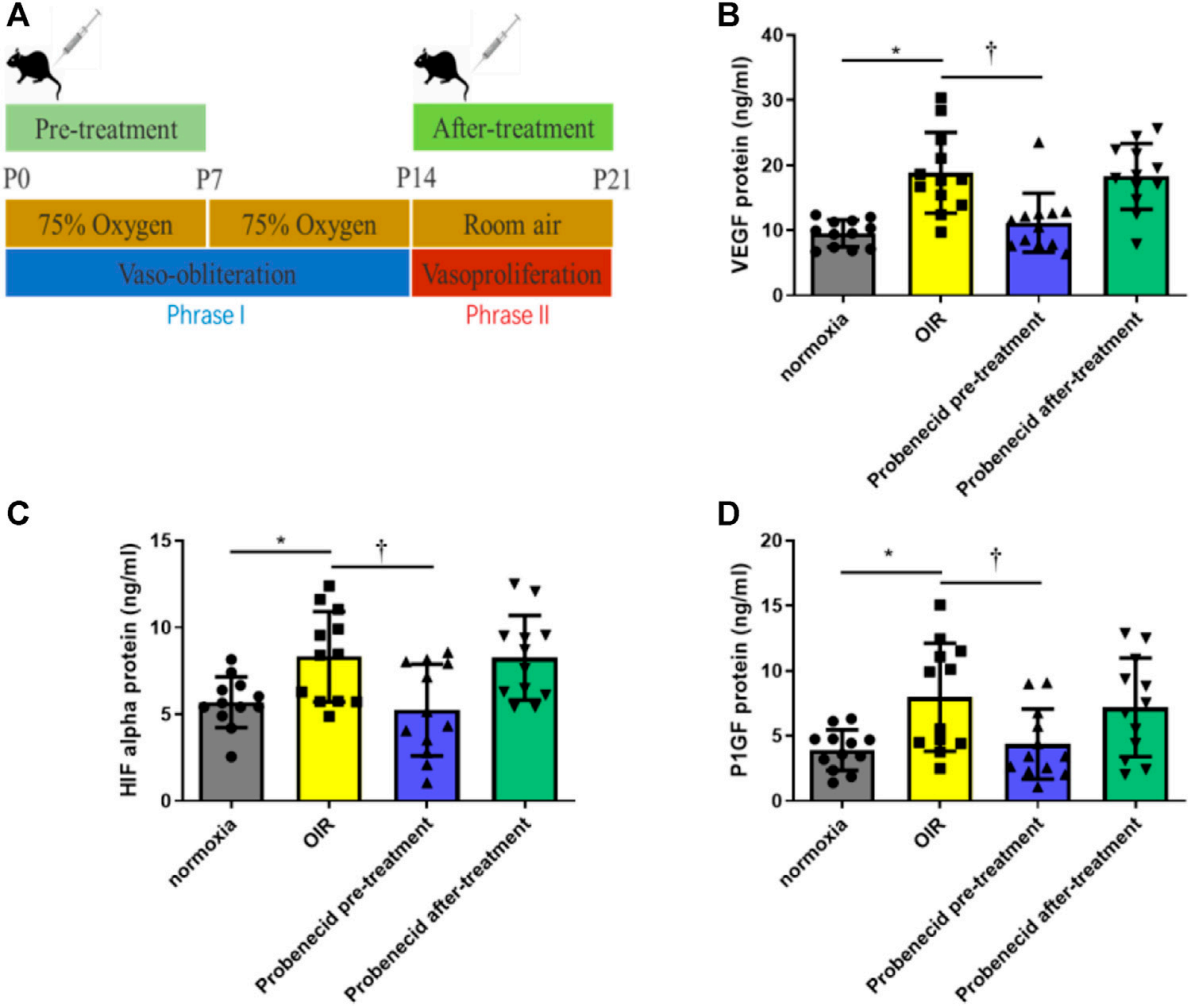
Probenecid inhibits overproduction of VEGF, HIF-1α, and P1GF induced by hypoxia. **(A)** Scheme of oxygen-induced retinopathy (OIR): SD rat pups are exposed to 75% oxygen from P1 to P14 to induce retinal vessel loss and are returned to room air at P15–P21. ELISA was used to evaluate **(B)** VEGF, **(C)** HIF-1α, and **(D)** P1GF levels in tissue lysates. Bar graphs represent the concentrations of each protein in the four groups. Results are expressed as mean ± SD; n = 12 rats per group, *p < 0.05, vs normoxia; †*p* < 0.05, vs OIR. Significance was determined by one-way ANOVA with Tukey’s post hoc test.

Phase II ROP is characterized by increased levels of VEGF, HIF-1α, and PlGF due to peripheral avascular retinal hypoxia and metabolism demand augmentation. In this study, probenecid (1 mg/kg) pretreatment was performed i.p. once a day from P1 to P7 in newborn SD rats that were raised in 75% oxygen from P1 to P14. The vitreous humor and retina were harvested on P22 for VEGF, HIF-1α, and PlGF analyses. Compared with normoxic condition, OIR resulted in significant increases in VEGF, HIF-1α, and PlGF. We observed a significant decrease in the levels of VEGF, HIF-1α, and PlGF in the pretreated group as compared to the OIR pup group(*p* < 0.05). However, in the after-treatment group, when the OIR rats had returned to the normoxic condition from P15 to P21, no decrease in VEGF, HIF-1α, or PlGF was detected, and the levels were similar to those of the OIR animals (Figure 2).

### Probenecid Attenuated Oxygen Fluctuation–Induced Retinal Neovascularization

**FIGURE 3:**
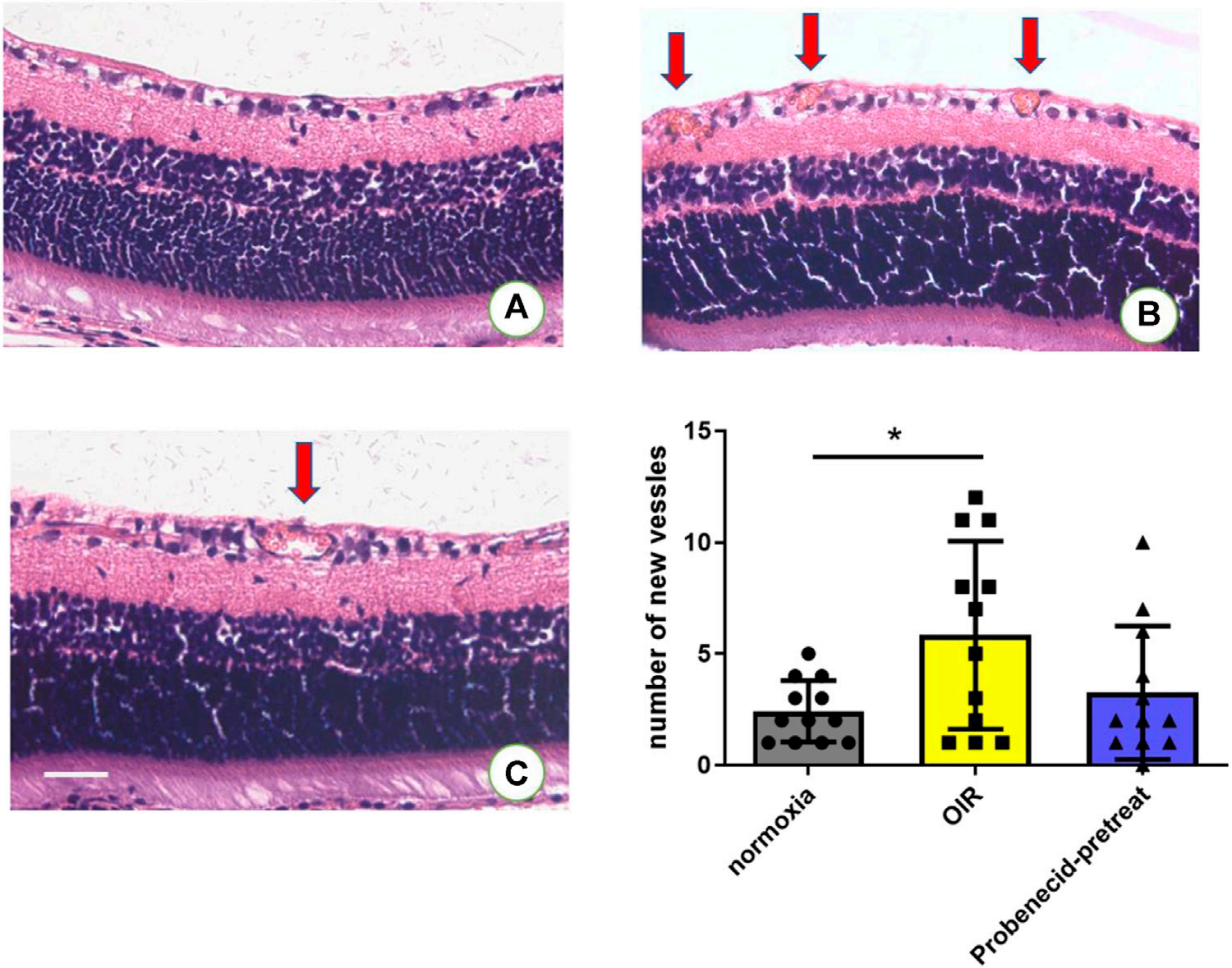
Probenecid treatment diminished retinal neovascularization in H&E-stained retinal sections in **(A)** normoxic groups and **(B)** OIR groups. **(C)** Probenecid pretreatment was used for the retinal neovascularization analysis. No significant difference in the average number of pre-retinal vascular lesions was observed between normoxic and probenecid pretreatment retinas on P22 (n = 12 per group). Kruskal–Wallis test. Scale bar: 20 μm.

Neovascularization is characterized by the development of sprouts from retinal vessels. These newly formed sprouts penetrate the inner limiting membrane (ILM) in most cases. In OIR rats, probenecid (i.p., 1 mg/kg) was administered from P1 to P7 (Figure 1A). H&E staining was used to compare the newly formed intravitreal retinal vessels (arrows, Figure 3) in different groups. Exposure to 75% oxygen from P1 to P14 and return to normoxic conditions from P15 to P21 resulted in a drastic increase in the number of neovascular endothelial cells that penetrated the ILM. This progression of vascularization was attenuated if the mice were pretreated with probenecid.

### Probenecid Decreased the Central Vaso-Obliteration Area in OIR

**FIGURE 4:**
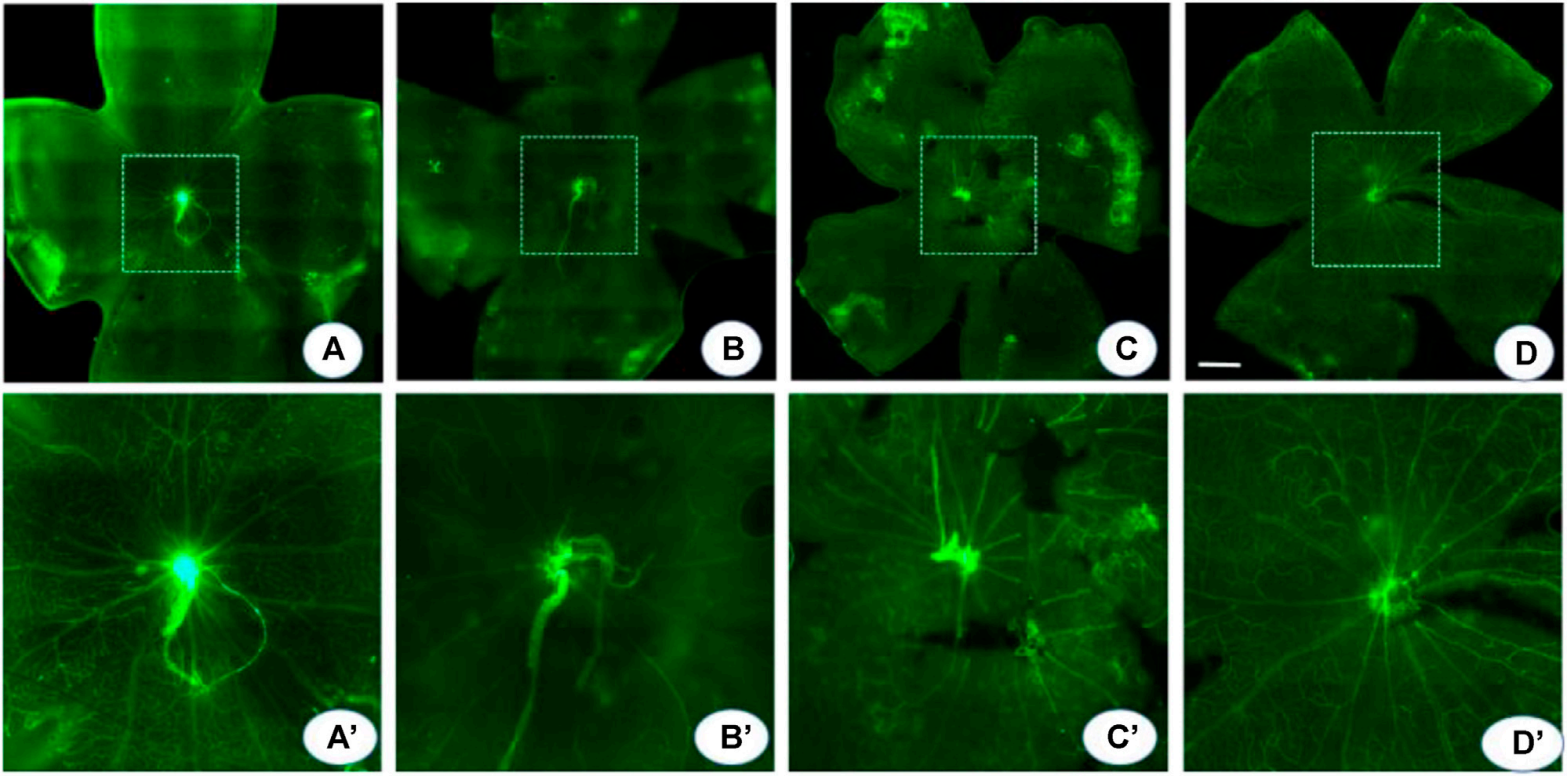
Probenecid decreased central vaso-obliteration. An isolectin fluorescein–stained retinal flat mount was used to detect the central vaso-obliterated area. **(A)** In the normoxia group, the retinal blood vessels are arranged radially with the optic disc as the center, and the quadrants are evenly and symmetrically distributed. The blood vessels run naturally without any disorder or twist. **(B, C)** OIR and dH_2_O-treated controls show a vaso-obliteration area in the posterior pole with clump-shaped capillary leakage. In the border zone, the large branch of the blood vessel becomes thin and tortuous. The distorted small branch blood vessel is twisted and clumped. There is extensive neovascularization in the whole retina, local vasodilation, and fluorescein infiltration. **(D)** Probenecid partially alleviated these pathological changes. **(A′-D′)** are montages of the **(A-D)** central area in dash. *p* < 0.05 (normoxia vs OIR, normoxia vs OIR + dH_2_O, and OIR vs OIR + probenecid), one-way ANOVA with Tukey’s post hoc test. Scale bar: 500 μm.

In the normoxic conditions, retinal blood vessels were distributed in a uniform network structure with no non-perfusion areas. In contrast, in OIR mice, a large area of avascularity indicating vaso-obliteration was observed in the center of the retina, and emerging neovascular buds were seen at the junction of the non-perfusion area and the perfusion area. Compared to OIR mice, probenecid-treated mice displayed significantly smaller central retinal vaso-obliterated areas (Figure 4). The relative vessel obliteration area in normoxic OIR, OIR + dH_2_O, and OIR + probenecid was 23.04 ± 5.20%, 41.67 ± 7.71%, 38.69 ± 10.44%, and 30.08 ± 6.42%, respectively (Figure 4). Hypoxia stimulates angiogenesis in vascular and non-vascular sections of the eye, resulting in neovascularization and ROP. For flat-mounted retinas, a significantly reduced neovascular area was found in the probenecid-treated retinas versus the dH_2_O-treated controls.

### Oxygen Fluctuation–Induced Increase in Hepcidin Was Suppressed by Probenecid

**FIGURE 5:**
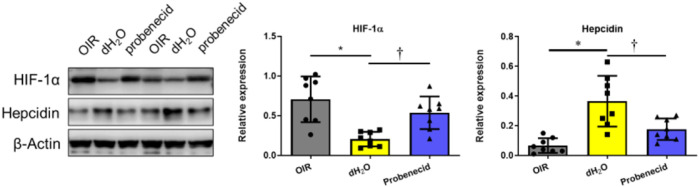
Probenecid inhibits overproduction of hepcidin induced by hyperoxia in OIR rats were treated with probenecid or an equivalent volume of dH_2_O from P1 to P7. Representative bands from two different samples are shown. Bar graphs represent the relative expression level of HIF-1α and hepcidin. Results are expressed as mean ± SD; n = 8 per group, *p < 0.05 vs the control group; †*p* < 0.05 vs O_2_ + dH_2_O. Significance was determined by one-way ANOVA with Bonferroni’s multiple comparisons test.

At P22, liver tissue was harvested and the expression levels of HIF-1α and hepcidin, a cationic peptide hormone produced by hepatocytes involved in iron metabolism regulation, were examined by Western blotting. We found that HIF-1α levels were significantly elevated in the livers of probenecid-treated OIR rats compared with sham-treated (dH_2_O) ones. On the contrary, while the hepcidin expression was markedly elevated in the OIR model, probenecid treatment significantly prevented the oxygen fluctuation–mediated increase in hepcidin (Figure 5).

## Discussion

In the present study, retinal EC proliferation, neovascularization, and central obliteration were observed in a murine model of OIR. Based on the CMap database, probenecid was implicated in the disrupted gene expression profile of retinal ECs during hypoxic conditions. Here, we demonstrate for the first time that probenecid treatment during exposure to high concentrations of oxygen was associated with reduced severity of these pathological characteristics. These findings suggest a potential therapeutic role of probenecid in ischemia-induced retinopathy.

Experimental and clinical evidence demonstrates that fluctuations in oxygenation are associated with the development of severe ROP. The murine model of fluctuating oxygen is most representative of human ROP (Hartnett and Penn, 2012). Vähätupa et al. recently used the OIR mouse model to discover new therapeutic drug targets in retinopathies using a novel mass spectrometric technique and sequential window acquisition of all theoretical fragment ion mass spectra in genetically modified mouse strains (Vahatupa et al., 2020). ROP is almost exclusively a disease found in premature infants. In rats, there is little retinal vascular development until birth. The deep capillary plexus, which develops into veins, begins growing by day 9 and is complete by day 15. Yielding a human-like pattern of retinal neovascularization, we applied a two-stage OIR model in rats which are similar to those used in the clinical setting.

The typical pathogenesis of ROP consists of two distinct phases: a vaso-obliterative phase, which begins immediately after birth due to oxygen levels being higher than those *in utero*, and vasoproliferative phases, which are marked by the overproduction of VEGF, especially in the presence of retinal hypoxia. During the latter phase, low levels of antioxidants in the retina lead to oxygen-mediated lipid peroxidation with the release of active nitric oxide; this, in turn, results in impaired retinal circulation and vascular integrity, leading to vasoproliferation. Understanding the two phases of ROP is critical for identifying appropriate therapeutic strategies and improving visual outcomes in prematurity. The successful treatment of a threshold of ROP relies on multiple factors. In addition to laser or cryotherapy as standard care, new therapeutic options targeting the pathogenesis of ROP are emerging. Although no FDA-licensed antagonists of VEGF are available for use in prematurity, intravitreal bevacizumab showed promising results for zone I or zone II posterior stage 3 + ROP, with a portion of non-responsive patients and numerous unwanted side effects (Yang et al., 2016). As the β-adrenoreceptor pathway is involved in hypoxia-induced vasoproliferation (Buhrer et al., 2018), oral propranolol and eye drops are currently being tested (CTRI/2013/11/004131, NCT02014454). Erythropoietin (EPO) is another hypoxia-induced factor that induced angiogenesis *in vitro* to a similar extent as VEGF. The effect of EPO on ROP depends on the timing of administration; it may reduce vaso-obliteration if administered during phase I ROP, whereas it appeared to increase the risk of ROP at any grade in a previous retrospective study (Kandasamy et al., 2014). Several other drugs have been tested with inconclusive results, such as D-penicillamine, vitamins A and E, and omega-3 PUFA supplementation. The optimal molecular mechanism for future anti-angiogenic therapies is one where the angiogenic blood vessels are “normalized” to alleviate hypoxia and inhibit the detrimental aberrant vascular leakage, which leads to fiber proliferation.

Insufficient knowledge of the interactions of retinal development, injury, and repair pathways and oxidative stress hampers the development of effective treatments. An essential challenge that arises throughout biomedicine is the need to establish a network between diseases, physiological processes, and the action of small-molecule therapeutics. With regard to ROP, much of the understanding has come from clinical observations, followed by animal studies to determine pathogenesis, which ultimately leads to clinical intervention studies and subsequent changes in practice. This endeavor continues to refine patient care but also proves to be both time-consuming and expensive. Unlike customary approaches, Bucolo, C. and colleagues used a bioinformatics analysis with access to Gene Expression Omnibus dataset and GENEMANIA-Cytoscape, an enrichment of the information approach to identify a focused set of miRNAs and miRNA–mRNA interactions that might be pharmacological targets and biomarkers for diabetic retinopathy diagnosis and treatment (Platania et al., 2019). In the current study, we used CMap to identify a candidate agent for early ROP and validated its potential as a therapeutic agent for angioneoplastic retinopathy.

CMap helps develop compounds based on the connection of genes, drugs, and disease states by virtue of common gene expression signatures. By inferring the main chemical structure of small molecules, it is possible to predict potential and unexpected mechanism directions of target molecules. Given the debate about the limitations and low time efficiency of synthetic new drugs, CMap is considered to be a beneficial tool for identifying new applications for establishing drugs. Lee et al. reported a natural compound from *Securinega suffruticosa* securinine, which was identified as a vascular protective agent targeting atherosclerosis in vascular endothelial cells and smooth muscle cells (Lee et al., 2021). Indeed, atractyloside was determined to be a potential drug candidate for type 2 diabetes based on CMap analysis. Moreover, Zhu et al. identified a list of potential drug candidates for repositioning by deriving disease signatures from the transcriptomic profiles of human-induced pluripotent stem cells (iPSCs) from patients with Noonan and LEOPARD syndrome, and reverse-correlated these signatures to drug transcriptomic signatures from CMap and L1000 projects (Zhu et al., 2020).

In our study, overproduction of PlGF in phase II ROP was prevented by probenecid pretreatment. PlGF is a pleiotropic cytokine, similar to VEGF, which binds to its membrane-bound receptor—fms tyrosine kinase 1 (Flt-1)—to stimulate angiogenesis. Studies have shown that the levels of VEGF-A, VEGF-B, and PlGF are elevated in ROP. Exogenous PlGF stimulated cell proliferation and migration under hypoxic conditions, whereas PIGF expression was reversed by anti-VEGF therapy in the OIR model. Anti-PlGF treatment was effective for neovascular tufts in OIR mice; hence, it could be used as a marker for disease prognosis and combined therapy (Mesquita et al., 2018).

Hepcidin, a master iron sensor, is a protein found in humans that is encoded by the hepcidin antimicrobial peptide (HAMP) gene. In addition to fine-tuning systemic iron trafficking, hepcidin is also localized in tissues, including the brain, suggesting that the extrahepatic pool of hepcidin is instrumental in locally regulating tissue iron homeostasis. Hepcidin transcription is activated by ferritin saturation or inflammation. Ferroportin is degraded by hepcidin, resulting in the reduction of iron export. Cardiomyocyte-specific deletion of hepcidin leads to long-term heart dysfunction (Lakhal-Littleton et al., 2016). Here, we demonstrated that the administration of probenecid prior to hyperoxia-abrogated hepcidin secretion. Notably, hepcidin production is also regulated by inflammatory cytokines, such as IL-6 (Zlatanova et al., 2019). Increasing evidence suggests that the IL-6 pathway plays a prominent role in ROP pathogenesis and that interactions between IL-6 and endothelial cells regulate the recruitment of leukocytes and the expression of inflammatory proteins (Bartkeviciene et al., 2020; Woo et al., 2020). Clinically, anemia is common in prematurity, and repeated red blood cell infusion and accompanying iron overload are risk factors for ROP (Aher and Ohlsson, 2019). The present results support the hypothesis that dysregulation of iron homeostasis genes plays an important role in the modulation of vascular homeostasis. Further research on the regulation of iron homeostasis biomarkers such as hepcidin and ferroportin will be required in the regression of retinal neovascularization.

Our study repurposes a well-known drug for a rare disease, using microarray technology and hypoxic conditions, and reported that dysregulation of genes involved in iron homeostasis-mediating oxidative damage may be responsible for the mechanisms underlying ROP. However, the use of probenecid needs further investigation as our study has some limitations. First, the identification of the compound was based on gene expression information from cell cultures, which are not always indicative of the biological effects observed in human tissues, and the pathways involved and gene regulation in complex diseases such as retinopathy of prematurity were not fully taken into consideration. Second, only gene expression information was considered in CMap, while information about gene regulation and modules/pathways was largely ignored. Third, transcription factor expression itself does not usually reflect changes in activity due to posttranscriptional modifications and other complications, especially when bipolar disorders, such as ROP, are involved. A module-oriented connectivity map approach using transcription factor–centered networks would aid the query for new repositioning candidates.

## Conclusion

Our findings suggest that the CMap approach is promising for drug repositioning. Probenecid prohibits retinal EC proliferation and angiogenesis and prevents overproduction of VEGF and HIF-1α in phase II ROP. Probenecid may mediate its effects by inhibiting hepcidin/HAMP overexpression.

## Data Availability

The raw data supporting the conclusions of this article will be made available by the authors, without undue reservation.
